# Enhanced Magnetic and Dielectric Performance in Fe_3_O_4_@Li_0.5_Cr_0.5_Fe_2_O_4_ Core/Shell Nanoparticles

**DOI:** 10.3390/nano15141123

**Published:** 2025-07-19

**Authors:** Mohammed K. Al Turkestani

**Affiliations:** Department of Physics, Umm Al-Qura University, Mecca 24381, Saudi Arabia; mkturkestani@uqu.edu.sa

**Keywords:** core/shell, magnetic, sol–gel, dielectric properties

## Abstract

This study presents the first successful integration of Fe_3_O_4_ and Li_0.5_Cr_0.5_Fe_2_O_4_ into a well-defined core/shell nanostructure through a two-step synthesis that combines co-precipitation and sol–gel auto-combustion methods. Unlike conventional composites, the core/shell design effectively suppresses the magnetic dead layer and promotes exchange coupling at the interface, leading to enhanced saturation magnetization, superior magnetic heating (specific absorption rate; SAR), and improved dielectric properties. Our research introduces a novel interfacial engineering strategy that simultaneously optimizes both magnetic and dielectric performance, offering a multifunctional platform for applications in magnetic hyperthermia, electromagnetic interference (EMI) shielding, and microwave devices.

## 1. Introduction

In recent years, core/shell nanostructures have emerged as an effective design strategy for tailoring and enhancing the magnetic and dielectric properties of nanomaterials, particularly in the realm of spinel ferrites. Ferrites, with the general formula MFe_2_O_4_ (where M = Fe, Co, Ni, Zn, Mn, etc.), exhibit a unique combination of magnetic, electrical, and dielectric behaviors due to their mixed valency and cation distribution within the tetrahedral (A) and octahedral (B) sites of the spinel lattice [1,2]. These materials find extensive applications across various fields, including magnetic data storage, biomedical devices, catalysis, electromagnetic wave absorption, EM shielding, high-frequency communication components, and microwave devices [3,4,5,6]. However, when ferrites are reduced to the nanoscale, their physical properties deviate significantly from those of their bulk counterparts due to an increased surface-to-volume ratio, the presence of surface defects, and finite-size effects. Notably, the magnetic behavior is impacted by surface spin disorder, leading to reduced saturation magnetization and the emergence of so-called “magnetic dead layers” [7]. Similarly, their dielectric properties are governed by interfacial polarization and grain boundary contributions, which are highly sensitive to particle size, morphology, and structural heterogeneity [8]. The core/shell configuration has gained substantial attention in addressing these limitations and further optimizing material performance. In a typical core/shell structure, a ferrimagnetic core is coated with another ferrite phase, oxide, or non-magnetic material. This architecture introduces interfacial effects that significantly influence magnetic anisotropy, exchange coupling, and electrical insulation, thereby enhancing the desired properties of the material [9].

The magnetic properties of core/shell ferrites are governed by several key parameters: core size, shell thickness, interfacial strain, and magnetic exchange interactions between the core and shell materials. For instance, Fe_3_O_4_/ZnFe_2_O_4_ core/shell nanoparticles exhibited enhanced coercivity and reduced saturation magnetization due to spin pinning at the core/shell interface [10]. Similarly, CoFe_2_O_4_/Fe_3_O_4_ core/shell nanoparticles demonstrated increased magnetic anisotropy and tunable exchange bias, characteristics that are beneficial for high-density magnetic storage and spintronic devices [11]. Moreover, the presence of a magnetically “hard” shell over a “soft” magnetic core, or vice versa, can lead to exchange-spring behavior, wherein magnetic reversal is controlled through interfacial exchange coupling [12]. This principle has been exploited in systems like NiFe_2_O_4_/CoFe_2_O_4_ and MnFe_2_O_4_/Fe_3_O_4_ core/shell structures, where the core/shell interface plays a crucial role in modulating coercivity and remanence through exchange interactions [13].

The dielectric properties of ferrite-based nanomaterials are equally sensitive to microstructural features such as grain boundaries, lattice defects, and phase segregation. In core/shell structures, the interface between the core and shell phases acts as a significant contributor to interfacial polarization, thereby enhancing the dielectric constant and minimizing dielectric loss, especially at high frequencies [14]. For example, TiO_2_-coated NiZn ferrites demonstrated improved dielectric stability and reduced eddy current loss, a benefit attributed to the insulating nature of the shell [15]. Similarly, spinel ferrite systems like MnFe_2_O_4_/SiO_2_ or CoFe_2_O_4_/BaTiO_3_ exhibited enhanced dielectric constants, which are attributed to Maxwell–Wagner-type interfacial polarization mechanisms and the suppression of leakage current through shell insulation [16]. These characteristics are crucial for applications in high-frequency devices, microwave absorbers, and energy storage systems. The core/shell strategy offers multiple advantages over conventional single-phase or composite structures, such as enhanced stability—where the shell can protect the magnetic core from oxidation, agglomeration, or structural degradation—and interfacial tuning, where the interface serves as a zone for manipulating exchange bias, spin pinning, or charge distribution. Furthermore, core/shell architectures can provide synergistic effects for multifunctional applications, such as magneto-dielectric coupling, magnetic hyperthermia, or multiferroic behavior [17].

In this context, we have chosen to synthesize a core/shell structure based on Fe_3_O_4_ as the core and Li_0.5_Cr_0.5_Fe_2_O_4_ as the shell. Fe_3_O_4_ is a well-known soft magnetic material known for its high saturation magnetization and good conductivity, while Li-Cr ferrites exhibit semiconducting properties and structural stability, albeit with reduced magnetic activity. This unique combination allows us to investigate the role of a magnetically dilute shell in modifying the magnetic profile of the core, particularly through the formation of a magnetic dead layer at the interface. Moreover, the electrical resistivity of the shell may help to enhance the dielectric performance by suppressing conduction losses, thereby improving frequency response and insulating capabilities. This tailored architecture is intended to demonstrate how a weak magnetic shell can effectively modulate both magnetic saturation and dielectric loss, fulfilling dual functions in multifunctional magnetic–dielectric devices. By comparing this core/shell structure with both individual and composite systems, this work highlights the distinct advantages of coherent interfaces over random physical mixtures. Such an approach paves the way for advanced material designs for spintronic, magnetoelectric, and high-frequency applications.

## 2. Materials and Methods

### 2.1. Synthesis of Fe_3_O_4_ Nanoparticles

Magnetite (Fe_3_O_4_) nanoparticles were synthesized using a standard co-precipitation technique, renowned for its ability to produce fine, phase-pure ferrite powders under mild conditions. In this process, ferric nitrate [Fe(NO_3_)_3_·9H_2_O] and ferrous nitrate [Fe(NO_3_)_2_·6H_2_O] were used as the sources of Fe^3+^ and Fe^2+^ ions, respectively. Both salts were of analytical grade, boasting a purity of 99.9%, and were procured from Merck.

To initiate the synthesis, a stoichiometric ratio of Fe^3+^ to Fe^2+^ (2:1) was dissolved in deionized water while maintaining continuous stirring at 60 °C, resulting in a clear brown solution. The pH of this solution was subsequently elevated to approximately 10 through the dropwise addition of ammonium hydroxide (NH_4_OH), which facilitated the formation of a black precipitate of Fe_3_O_4_. The mixture was stirred for an additional hour to ensure complete precipitation. The resulting precipitate was then separated using a magnetic field, thoroughly washed multiple times with deionized water and ethanol to remove residual ions, and dried overnight at 80 °C. The dried powder was finally ground into fine particles for further use.

### 2.2. Synthesis of Li_0.5_Cr_0.5_Fe_2_O_4_ Ferrite

The Li–Cr substituted ferrite with the nominal composition Li_0.5_Cr_0.5_Fe_2_O_4_, which was prepared via the sol–gel auto-combustion method—a technique that offers the advantage of achieving homogeneous mixing at the atomic level. High-purity nitrate salts, including lithium nitrate [LiNO_3_], chromium nitrate [Cr(NO_3_)_3_·9H_2_O], and ferric nitrate [Fe(NO_3_)_3_·9H_2_O] (all sourced from Merck with 99.9% purity) were used as starting materials.

The appropriate stoichiometric amounts of these nitrates were dissolved in a minimal volume of deionized water and stirred continuously at 80 °C to create a uniform solution. Citric acid was added as a chelating agent in a 1:1 molar ratio with respect to the total metal ions. The solution was stirred until a transparent gel was formed. Upon further heating, the gel underwent spontaneous ignition due to the exothermic redox reaction between nitrates and citric acid, yielding a voluminous and fluffy powder. The obtained powder was then calcined at 600 °C for three hours to promote crystallization of the ferrite phase.

### 2.3. Fabrication of Core/Shell (Fe_3_O_4_@Li_0.5_Cr_0.5_Fe_2_O_4_) Nanostructure

The core/shell nanostructure was synthesized by coating a shell of Li_0.5_Cr_0.5_Fe_2_O_4_ over the Fe_3_O_4_ cores through a modified sol–gel auto-combustion route. For this purpose, 1 g of previously synthesized Fe_3_O_4_ powder was dispersed in the aqueous precursor solution of Li, Cr, and Fe nitrates, formulated to yield 0.25 g of the Li_0.5_Cr_0.5_Fe_2_O_4_ shell. The dispersion underwent vigorous stirring and mild heating (80 °C) to ensure uniform deposition of the gel matrix on the Fe_3_O_4_ surface. Citric acid was added as the complexing agent, facilitating the formation of a viscous gel. Upon further heating, the gel underwent auto-combustion, resulting in a fine powder that encapsulated the Fe_3_O_4_ core within a Li–Cr ferrite shell. The final product was annealed at 600 °C for three hours to enhance interfacial adhesion and crystallinity.

## 3. Results and Discussion

### 3.1. X-Ray Diffraction (XRD) Analysis

**Figure 1:**
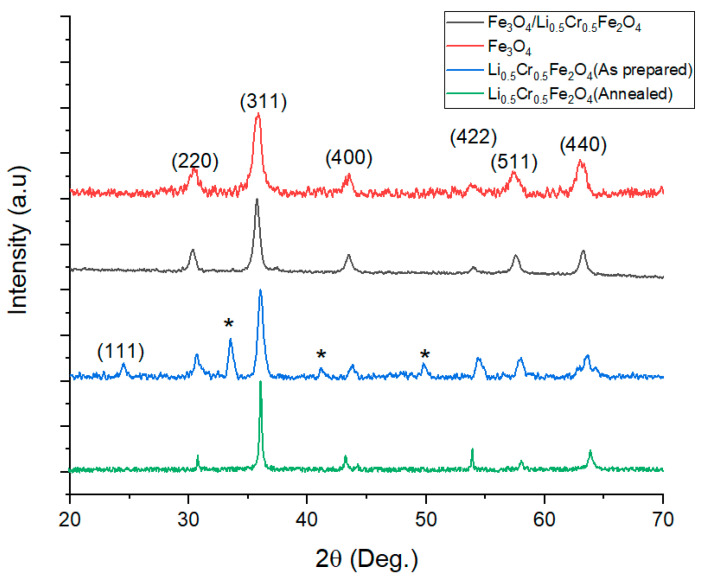
XRD chart for the prepared samples. The asterisk peaks indicate the presence of a β-LiFe_2_O_4_ phase.

The X-ray diffraction patterns of Fe_3_O_4_, Li_0.5_Cr_0.5_Fe_2_O_4_, and the core/shell Fe_3_O_4_@Li_0.5_Cr_0.5_Fe_2_O_4_ samples are presented in Figure 1. Both the Fe_3_O_4_ and core/shell Fe_3_O_4_@Li_0.5_Cr_0.5_Fe_2_O_4_ samples exhibit well-defined diffraction peaks characteristic of the cubic spinel structure, consistent with JCPDS cards No. 01-071-6336 and No. 38-0259. In addition, the Li_0.5_Cr_0.5_Fe_2_O_4_ (as prepared) sample displays extra reflections at 2θ = 33.5°, 41.1°, and 49.8°, which can be attributed to the presence of a secondary β-phase related to the LiFe_2_O_4_ structure. This phase typically forms under low-temperature synthesis conditions (up to 600 °C). Upon annealing at 850 °C for 3 h, these secondary peaks disappear, indicating the transformation into a single-phase spinel structure. The most intense diffraction peak at 2θ ≈ 35.88°, corresponding to the (311) plane, confirms the presence of the spinel phase across all samples. The calculated lattice parameters for Fe_3_O_4_, Li_0.5_Cr_0.5_Fe_2_O_4_, and the core/shell sample are 8.325 Å, 8.275 Å, and 8.319 Å, respectively. The slight decrease in the lattice constant for Li_0.5_Cr_0.5_Fe_2_O_4_ compared to Fe_3_O_4_ is attributed to the partial substitution of Fe^3+^ ions (ionic radius ≈ 0.645 Å) with Cr^3+^ (≈ 0.615 Å) and Li^+^ (≈0.76 Å), influencing the lattice structure depending on the cation distribution and site occupancy [18].

To quantitatively evaluate the degree of lattice deformation at the core–shell interface, the lattice mismatch ratio (f) is calculated as follows:

Using a_core_ = 8.325 Å (Fe_3_O_4_) and a_shell_ = 8.287 Å (Li_0.5_Cr_0.5_Fe_2_O_4_), the lattice mismatch is

$$\frac{∆a}{a}=\frac{a_{shell}-a_{core}}{a_{core}}$$

$$\frac{∆a}{a}=\frac{8.275-8.325}{8.325}=-0.6\%$$

The negative sign indicates that the shell lattice is slightly contracted in relation to the core. This small mismatch supports coherent or semi-coherent growth of the shell around the core, resulting in minimal interfacial dislocations [19]. Crystallite size values, calculated using the Scherrer equation, were 11.9 nm for Fe_3_O_4_, 14.0 nm for Li_0.5_Cr_0.5_Fe_2_O_4_, and 16.7 nm for the core/shell sample. The observed increase in crystallite size within the core/shell structure suggests enhanced crystallinity and structural integrity at the interface [20]. Finally, the micro-strain (ε), estimated by the Williamson–Hall method, increased from 0.0012 in Fe_3_O_4_ to 0.0017 in Li_0.5_Cr_0.5_Fe_2_O_4_, ultimately reaching 0.0024 in the core/shell sample. The rise in micro-strain further corroborates the presence of the lattice-mismatched interface between the core and shell [21].

In summary, the simultaneous increase in crystallite size and micro-strain, along with a small lattice mismatch (~0.6%), confirms the successful formation of a coherent Fe_3_O_4_@Li_0.5_Cr_0.5_Fe_2_O_4_ core/shell nanostructure, with strain accommodation at the interface being a key structural feature.

### 3.2. FTIR Spectra Analysis

**Figure 2:**
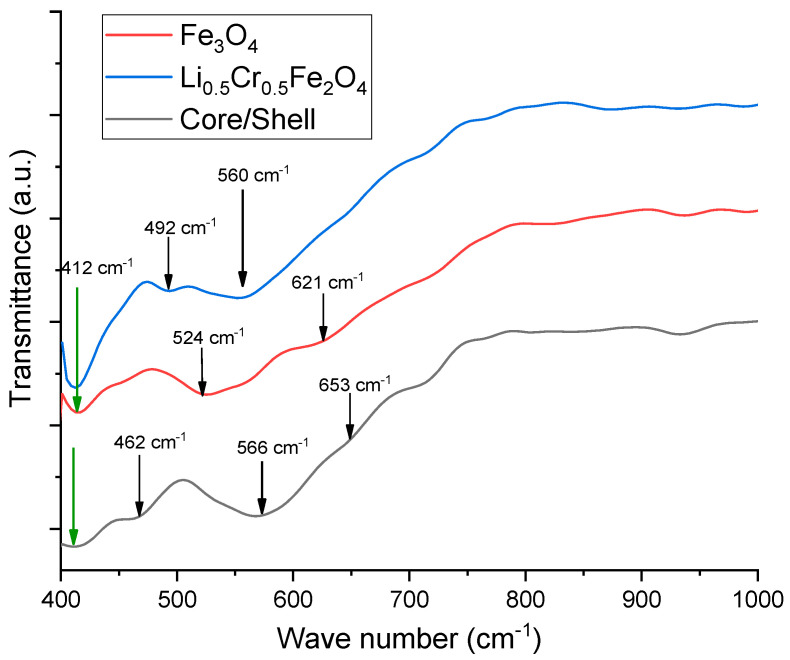
FTIR spectra for the prepared samples.

The Fourier Transform Infrared Spectroscopy (FTIR) spectra of the three samples (Figure 2) exhibit characteristic absorption bands of spinel ferrites in the range of 400 to 700 cm^−1^, which are associated with metal–oxygen vibrational modes at tetrahedral (A) and octahedral (B) sites [22,23]. Generally, the higher wavenumber band (around 600–650 cm^−1^) corresponds to the stretching vibrations of metal–oxygen bonds in tetrahedral sites (M–O in A site), while the lower wavenumber band (around 400–550 cm^−1^) is attributed to octahedral site vibrations (M–O in the B-site) [24].

In the analysis of the Fe_3_O_4_ sample, prominent peaks at 524 cm^−1^ and 621 cm^−1^ were identified, characteristic of Fe_3_O_4_, where Fe^3+^ occupies both tetrahedral and octahedral sites in an inverse spinel structure [22]. In contrast, the Li-Cr ferrite sample displays a noticeable shift in the tetrahedral band to a lower wavenumber (~492 cm^−1^) and a shoulder at 412 cm^−1^, indicating substitutional changes due to Li^+^ and Cr^3+^ ions. The lighter Li^+^ (ionic radius ~0.76 Å) and smaller Cr^3+^ (0.615 Å), when compared to Fe^3+^ (0.645 Å), impact the force constants and bond lengths, thereby leading to observable vibrational shifts [25].

The FTIR spectrum of the core/shell Fe_3_O_4_@Li_0.5_Cr_0.5_Fe_2_O_4_ sample exhibits broad and partially split absorption bands centered around 462, 566, and 653 cm^−1^. These features differ from the well-defined, sharp bands typically observed in the pure Fe_3_O_4_ and Li–Cr ferrite phases. The broadening and asymmetry of these vibrational modes are attributed to the overlapping of Fe–O stretching vibrations from both spinel phases, which occur in similar spectral ranges. Due to this spectral overlap, the individual vibrational signatures of the core and shell phases are not distinctly resolved. The presence of a pronounced and enhanced band around 653 cm^−1^ is particularly significant and may be associated with increased stiffness of tetrahedral Fe–O bonds, likely resulting from lattice strain and interfacial coupling at the core/shell boundary [26]. Such strain-induced effects are well known to cause frequency shifts and mode broadening in nanoscale ferrites.

Moreover, the nanoscale dimensions of the particles and the presence of a coherent interface introduce structural disorder and partial cation redistribution, further contributing to the loss of spectral resolution. This results in the emergence of a composite vibrational signature, rather than separate bands for each component phase. These observations are consistent with prior studies on core–shell and doped ferrite systems, where the FTIR response reflects a combined vibrational environment due to mode coupling and partial structural mixing [27].

Thus, the evolution of band shape and position across the samples provides strong vibrational evidence for the formation of a well-integrated core/shell architecture, with significant interfacial interaction between the Fe_3_O_4_ core and Li_0.5_Cr_0.5_Fe_2_O_4_ shell.

### 3.3. TEM Analysis

**Figure 3:**
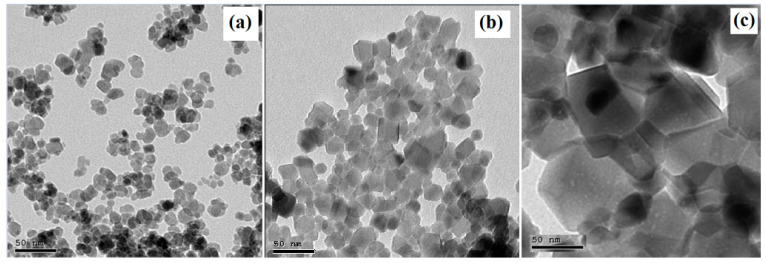
TEM images for the prepared samples (**a**) Fe_3_O_4_, (**b**) Li_0.5_Cr_0.5_Fe_2_O_4_, and (**c**) core/shell.

Transmission electron microscopy (TEM) micrographs of the three synthesized samples are depicted in Figure 3a–c. The morphological analysis of all samples reveals well-dispersed nanoparticles that exhibit shapes ranging from nearly spherical to polygonal. The estimated particle sizes for Fe_3_O_4_ (Figure 3a), Li_0.5_Cr_0.5_Fe_2_O_4_ (Figure 3b), and the core/shell Fe_3_O_4_@Li_0.5_Cr_0.5_Fe_2_O_4_ (Figure 3c) are approximately 12–15 nm, 18–20 nm, and 30–35 nm, respectively. Notably, the observed particle size for the core/shell sample is significantly larger than that of the bare core or shell samples, providing strong evidence for the successful formation of a shell layer over the core, which is consistent with the successful synthesis of the core/shell structure.

Interestingly, the particle size observed in the core/shell sample exceeds the crystallite size (~16.7 nm) obtained from X-ray diffraction (XRD) analysis. This discrepancy suggests that each particle may consist of multiple coherently diffracting crystallites (grains). Such characteristics would not contribute to the XRD crystallite size but would remain visible in TEM images. This behavior is commonly observed in polycrystalline core/shell systems, where the shell serves to provide structural protection or functional enhancement [28].

It is also worth noting that the core and shell regions are not sharply distinguishable in Figure 3c, likely due to the low contrast between Fe_3_O_4_ and Li_0.5_Cr_0.5_Fe_2_O_4_, both of which have similar electron densities. Moreover, the coherent interface formed due to good lattice matching (as evidenced by minimal lattice mismatch and strain values) may contribute to a uniform contrast, complicating the visual separation of the core and shell. These TEM findings, in conjunction with the XRD-derived crystal size and strain data, strongly support the formation of a coherent core/shell nanostructure in sample (c).

### 3.4. Energy-Dispersive X-Ray Spectroscopy (EDX) Analysis

**Figure 4:**
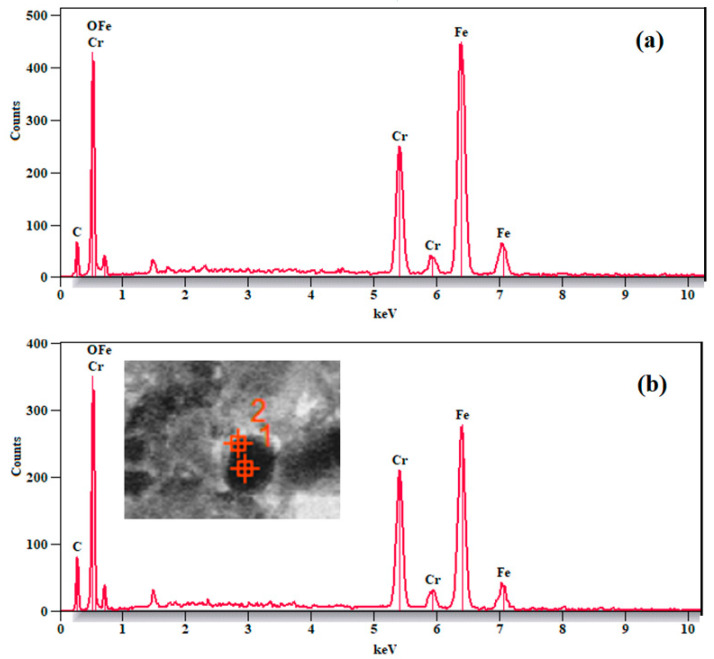
EDX measurement for (**a**) Fe_3_O_4_ and (**b**) core/shell samples.

To confirm the formation of the core/shell architecture, energy-dispersive X-ray spectroscopy (EDX) analysis was performed on two distinct regions of a single particle: the core (point 1) and the shell (point 2), as indicated in Figure 4b. The corresponding EDX spectra are presented in Figure 4a for the core and Figure 4b for the shell.

Quantitative analysis of the EDX spectra revealed the presence of Fe, Cr, and O elements in both regions, consistent with the expected composition of Fe_3_O_4_ and Li–Cr ferrite. However, the relative atomic concentrations of these elements varied significantly between the two locations, as summarized in Table 1.

In the core region (point 1), Fe was the dominant element (45.3 mol%), while Cr was present at a much lower concentration (12.3 mol%). This confirms that the core is primarily composed of Fe_3_O_4_. In contrast, the shell region (point 2) exhibited a markedly higher Cr content (27.1 mol%) with a reduced Fe concentration (30.6 mol%), indicating the shell is enriched in Cr, consistent with the composition of Li_0.5_Cr_0.5_Fe_2_O_4_.

These differences in elemental distribution between the core and shell provide strong evidence for a well-defined core/shell structure rather than a homogeneous mixture. Oxygen content remained relatively consistent in both regions (~42 mol%), reflecting its role in both oxide phases.

These findings substantiate the successful fabrication of a core/shell nanostructure, wherein the magnetic Fe_3_O_4_ core is encapsulated by a Li-Cr ferrite shell. This architectural design is particularly advantageous for dielectric and electromagnetic applications, as it enhances interfacial polarization and improves the overall dielectric response—a topic that will be further explored in the subsequent dielectric measurements.

### 3.5. Magnetic Characterization and Core/Shell Interface Effects

**Figure 5:**
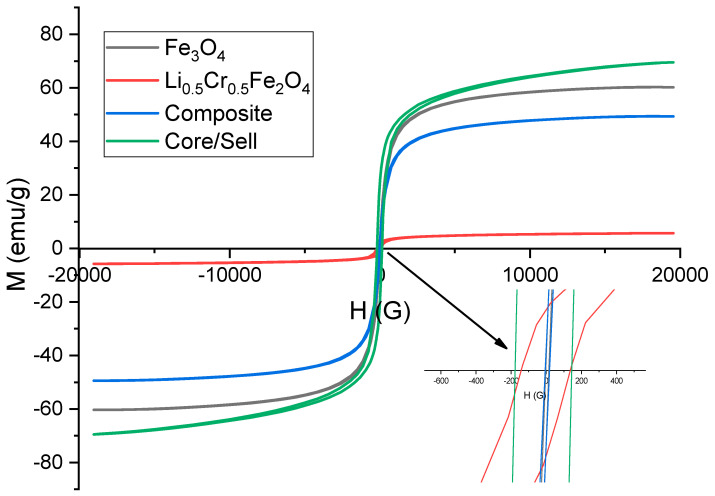
Magnetic hysteresis loops for the investigated samples.

The magnetic hysteresis loops at room temperature for the individual phases (Fe_3_O_4_ and Li_0.5_Cr_0.5_Fe_2_O_4_), their physical mixture (composite), and the core/shell structure are depicted in Figure 5. The inset figure shows the values of Hc of all the samples. It is obvious that all samples (except Li-Cr ferrite sample) have very small values of Hc, which exhibit super-paramagnetic behavior $\left(H_{c}\approx 0\right).$ This observation serves as a staunch indication of the formation of a magnetic phase at the nanoscale, especially for the Fe_3_O_4_ sample. The elevated coercivity (Hc) observed in the Li–Cr ferrite sample, compared to the other samples, can be attributed to the stronger spin–orbit coupling of Cr^3+^ ions (3d^3^) relative to Fe^2+^/Fe^3+^, which enhances the magnetocrystalline anisotropy of the system. On the other hand, the saturation magnetization (M_s_) values are approximately 60 emu/g for Fe_3_O_4_, 5 emu/g for Li_0.5_Cr_0.5_Fe_2_O_4_, 69 emu/g for the core/shell structure, and ~40 emu/g for the physical mixture (composite). The reduced saturation magnetization (Ms) of Li_0.5_Cr_0.5_Fe_2_O_4_ can be attributed to its cation distribution, where the substitution of non-magnetic Li^+^ ions at the B-sites leads to dilution of the magnetic sublattice. Additionally, the replacement of magnetic Fe^3+^ ions by Cr^3+^ ions at the B-sites further decreases the net magnetic moment, resulting in an overall reduction in magnetization.

As previously noted, the saturation magnetization of bare Fe_3_O_4_ nanoparticles is measured at 60 emu/g, which is considerably lower than that of the bulk material, typically around 92 emu/g [27]. This substantial reduction in magnetization at the nanoscale is primarily due to the increased surface-to-volume ratio and the pinning of surface spins resulting from the disruption of magnetic interactions at the nanoparticle surface [29]. Consequently, a magnetically inactive region, often referred to as a “dead layer,” forms at the surface, which consists of magnetic moments that do not contribute to the overall magnetization. The thickness of this layer can be estimated using a specific equation [30].

$$D_{eff}=D\sqrt[{3}]{\frac{M_{s(nano)}}{M_{s(bulk)}}}$$

where M_s_(nano) and M_s_(bulk) represent the saturation magnetization of the nanomaterial and the bulk material, respectively. D denotes the total physical diameter of the nanoparticle (12 nm), where D_eff_ refers to the effective magnetic diameter that actively contributes to magnetization. By utilizing this relationship, the effective magnetic diameter can be determined. Subsequently, the thickness of the magnetic dead layer can be estimated by the equation below [28]:

$$t=\frac{\left(D-D_{eff}\right)}{2}$$

From the equation presented above, the thickness of the magnetic dead layer was found to be around 1 nm (representing 23% of the total volume). The Li_0.5_Cr_0.5_Fe_2_O_4_ ferrite exhibits very low magnetization due to its inherently weak magnetic nature, confirming its role as a magnetically dilute shell material within the core/shell system. The composite sample, formed through the simple physical mixing of the two phases, demonstrates a linear magnetic response that aligns with the weighted average of the individual components, as anticipated. This observation confirms that there are no significant interfacial interactions or structure-induced magnetic modifications present in the composite sample [31].

In contrast, the core/shell structure displays a significantly higher saturation magnetization (69 emu/g) compared to the composite. This clearly indicates that the interface between the magnetic Fe_3_O_4_ core and the weakly magnetic shell plays a crucial role in preserving the magnetic integrity of the core [32]. In the context of core/shell structures, it is important to recognize that the total magnetization (M_s_) is not merely a linear sum of the individual magnetizations of Fe_3_O_4_ and Li_0.5_Cr_0.5_Fe_2_O_4_ (as observed in the composite scenario). Instead, the total magnetization of the core/shell system is determined using the following equation [33]:

$$M_{s}\left(Core/shell\right)=xM_{s}\left(Core\right)+yM_{s}\left(Shell\right)+M_{s}\left(int\right)$$

where x and y represent the weight ratio of the core/shell sample. The final term represents the nonlinear part of the magnetization, which reflects the change in magnetization resulting from the magnetic interaction at the interface.

Based on the magnetization data, the value of M_s_(int) is approximately 20 emu/g, which represents about 27% of the total magnetization of the sample. This enhancement can be attributed to the formation of a new spin periodicity at the surface of Fe_3_O_4_, which—combined with the magnetic moments of Li_0.5_Cr_0.5_Fe_2_O_4_ at the interface—may help in mitigating the effects of the magnetic dead layer in the following manner: in magnetic materials, the dead layer refers to a surface region where the magnetization is notably diminished or suppressed compared to the bulk. The creation of a core/shell structure between Fe_3_O_4_ and Li_0.5_Cr_0.5_Fe_2_O_4_ leads to a new configuration and periodic arrangement of spins that can restore or enhance magnetic ordering near the surface.

### 3.6. Magnetic Hyperthermia Performance and SAR Evaluation

**Figure 6:**
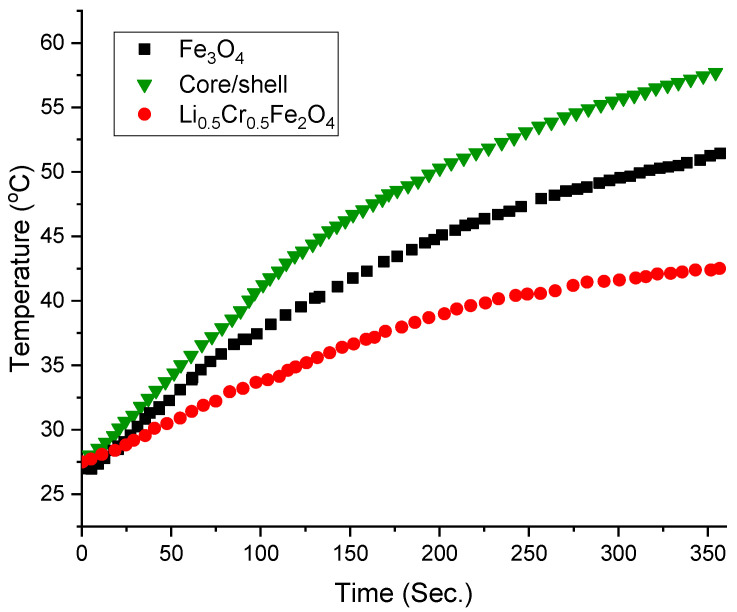
Temperature rise versus time under the effect of AC magnetic field.

The heating efficiency of magnetic nanoparticles is a key parameter in evaluating their suitability for magnetic hyperthermia applications. Figure 6 illustrates the temperature rise versus time curves for aqueous suspensions (10 mg/mL) of Fe_3_O_4_, Li_0.5_Cr_0.5_Fe_2_O_4_, and the Fe_3_O_4_/LiCr ferrite core/shell structure under an alternating magnetic field at H = 13 kA/m and a frequency of 120 KHz. All samples exhibit a clear and progressive increase in temperature over time, which is attributed to magnetic losses (primarily Néel and Brownian relaxation) in the presence of the AC magnetic field [34].

Among the three samples, the core/shell structure exhibits the highest heating rate, reaching a temperature of approximately 58 °C after 360 s, compared to 52 °C for pure Fe_3_O_4_ and only 43 °C for Li_0.5_Cr_0.5_Fe_2_O_4_. This enhanced thermal response directly reflects an increase in the specific absorption rate (SAR), calculated using the initial slope method and the following relation [35]:

$$SAR=\frac{C}{m}{\left(\frac{∆T}{∆t}\right)}_{initial}$$

where C is the specific heat capacity of the dispersion medium (water, 4.18 J/g·K), m is the mass concentration of the magnetic material (10 mg/mL), and (DT/Dt) is the initial slope of the temperature curve.

The estimated SAR values are presented below:

• Fe_3_O_4_: ~90 W/g;
• Li_0.5_Cr_0.5_Fe_2_O_4_: ~55 W/g;
• Core/Shell (Fe_3_O_4_@LiCr ferrite): ~120 W/g.

The significant improvement in SAR for the core/shell structure is ascribed to the reduction in the magnetic dead layer that typically forms at the surface of magnetic nanoparticles due to surface disorder and spin canting. The LiCr ferrite shell serves to passivate the surface of the Fe_3_O_4_ core, thereby restoring spin alignment at the interface and enhancing the effective magnetic moment. This leads to an improved magnetic response and energy dissipation under AC magnetic excitation. Consequently, the suppression of surface spin disorder along with the more coherent magnetic structure in the core/shell system are thus key factors in boosting SAR. Such enhancements are particularly valuable for biomedical applications like magnetic hyperthermia, where efficient thermal conversion at low particle concentrations is critical.

### 3.7. Dielectric Properties and Loss Tangent

To measure the dielectric constant and loss tangent, the powder is first pressed into a disk-shaped pellet with a known cross-sectional area (A) and thickness (t). The capacitance (C) and the phase angle (δ) are then measured at various frequencies.

The real part of the dielectric constant (ε) and the loss tangent (tan δ) are determined using the following equations:

$$C=\varepsilon \varepsilon_{o}\frac{A}{t}$$

$$Loss\;tangent=tan\left(\delta \right)$$

**Figure 7:**
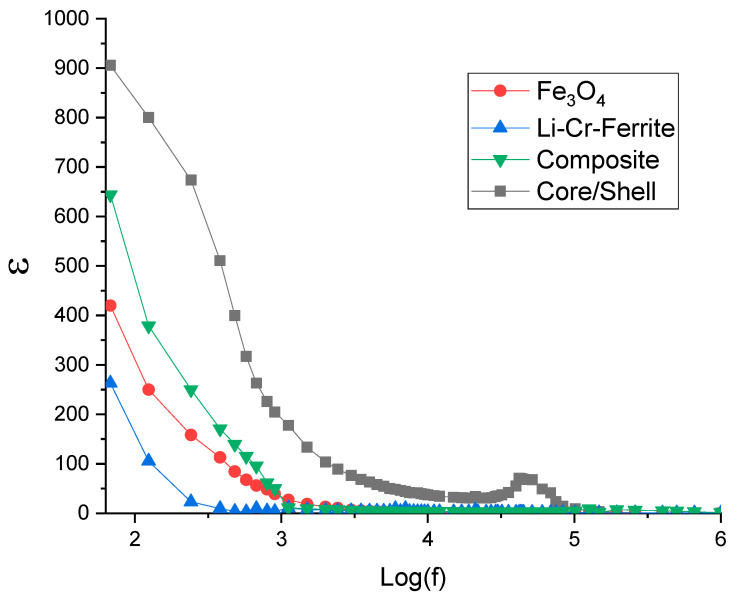
Real part of the dielectric constant versus frequency.

**Figure 8:**
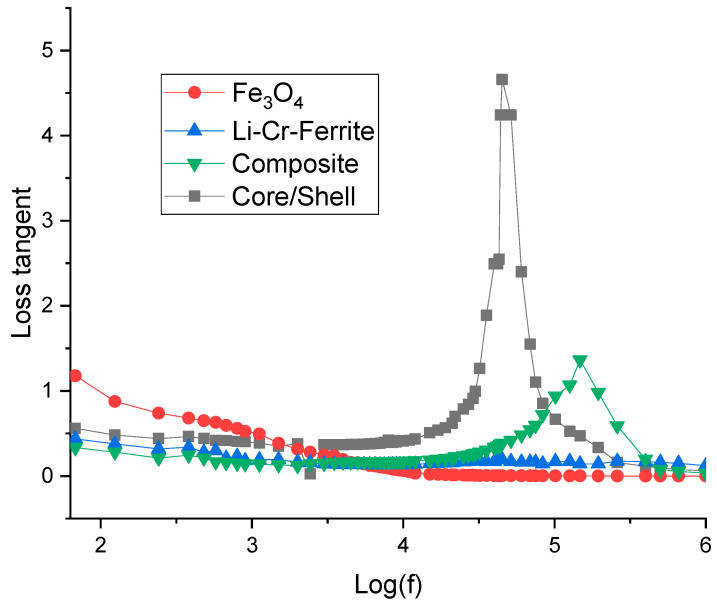
Loss tangent versus frequency.

The variation in the real part of the dielectric constant (ε) and dielectric loss tangent (tan δ) with log of base 10 of frequency—log(f)—for Fe_3_O_4_, Li-Cr ferrite, composite (Fe_3_O_4_ + Li-Cr ferrite), and core/shell samples is presented in Figure 7 and Figure 8. All samples exhibit a characteristic decrease in ε with increasing frequency, consistent with the Maxwell–Wagner interfacial polarization model and Koop’s phenomenological theory [36]. At low frequencies, charge carriers accumulate at grain boundaries, contributing to high polarization and, consequently, a large dielectric constant. As the frequency increases, the dipoles and charge carriers are unable to follow the rapidly oscillating electric field, resulting in a marked reduction in ε [37].

Among all samples, the core/shell structure exhibits the highest dielectric constant across the frequency range, reaching values near 900 at low frequencies, which significantly surpass both the individual components and their physical mixture (Composite). This enhancement can be attributed to the well-defined interface and improved space charge polarization at the core/shell boundary, which proves to be more effective than the loosely bound interface found in the physical mixture [38]. Furthermore, the enhancement in the dielectric constant relative to the core Fe_3_O_4_ sample can be attributed to the effects of Li and Cr ions. The incorporation of Li^+^ enhances ionic mobility, thereby promoting space charge polarization, while Cr^3+^ introduces localized electronic states that facilitate electron hopping, contributing to an increase in dielectric constant and a reduction in dielectric loss at low frequencies. In addition, lattice mismatch at the core–shell interface induces interfacial polarization, which further amplifies the overall dielectric response.

The dielectric loss tangent curves further validate this behavior. For the core/shell sample, a prominent relaxation peak appears around log(f) ≈ 4.6, which is absent or less pronounced in the other samples. This peak corresponds to a dielectric relaxation process, likely due to interfacial polarization or hopping conduction mechanisms that are amplified by the well-structured core/shell interface [39]. The composite sample demonstrates a broader and less intense peak at higher frequencies, suggesting a less effective polarization response due to non-uniform interfaces. In contrast, the Fe_3_O_4_ and Li–Cr ferrite samples exhibit relatively low and frequency-independent loss tangent values, suggesting minimal polarization or energy dissipation when evaluated individually [40]. Similar enhancements in dielectric loss have been previously reported for systems incorporating SiC@Fe_3_Si and C/SiO_2_ interfaces, where interfacial effects significantly contribute to increased polarization losses [41,42].

From an application perspective, the enhanced dielectric constant and tailored loss behavior of the core/shell structure render it an ideal candidate for high-performance energy storage devices, electromagnetic wave absorption, and microwave shielding. The ability to tune the interfacial characteristics in the core/shell design allows for the optimization of dielectric performance that cannot be achieved through mere physical mixing of components [43].

## 4. Conclusions

This study introduces a novel core/shell nanostructure, Fe_3_O_4_@Li_0.5_Cr_0.5_Fe_2_O_4_, synthesized through a hybrid co-precipitation and sol–gel auto-combustion route. The successful construction of a distinct core/shell configuration was confirmed by EDX elemental analysis, which verified the spatial separation of elements between the core and the shell. The core/shell structure demonstrated a substantial improvement in magnetic behavior—most notably, an increase in both saturation magnetization and specific absorption rate (SAR). These enhancements are attributed to the suppression of magnetic dead layers and the emergence of interfacial exchange coupling, which were absent in the physically mixed composite counterpart. Additionally, dielectric investigations revealed that the core/shell configuration supports a higher dielectric constant with reduced dielectric losses, underscoring its suitability for high-frequency applications, such as EMI shielding and microwave components. Overall, the novelty of this research lies in the rational design of a core/shell architecture that integrates and amplifies both magnetic and dielectric functionalities. The results provide new insights into tuning nanoscale interactions to achieve superior multifunctional performance in ferrite-based systems, establishing a promising foundation for future advanced magnetic and electronic materials.

## Figures and Tables

**Table 1:** Estimated elemental composition form EDX Spectra.

Region	Fe (mole%)	Cr (mole%)	O (mole%)
Core (Point 1)	45.3	12.3	42.5
Shell (Point 2)	30.6	27.1	42.4

## Data Availability

The original contributions presented in this study are included in the article. Further inquiries can be directed to the corresponding author.
